# Platelet-Activating Factor (PAF) Antagonistic Activity of a New Biflavonoid from *Garcinia nervosa* var. *pubescens* King

**DOI:** 10.3390/molecules170910893

**Published:** 2012-09-10

**Authors:** Juriyati Jalil, Ibrahim Jantan, Azura Abdul Ghani, Shahnaz Murad

**Affiliations:** 1 Drug and Herbal Research Centre, Faculty of Pharmacy, Universiti Kebangsaan Malaysia, Jalan Raja Muda Abdul Aziz, 50300 Kuala Lumpur, Malaysia; 2 Institute for Medical Research, Jalan Pahang, 50588 Kuala Lumpur, Malaysia

**Keywords:** *Garcinia nervosa* var. *pubescens*, biflavonoid, platelet-activating factor (PAF) antagonists, platelets

## Abstract

The methanol extract of the leaves of *Garcinia nervosa* var. *pubescens* King, which showed strong inhibitory effects on platelet-activating factor (PAF) receptor binding, was subjected to bioassay-guided isolation to obtain a new biflavonoid, II-3,I-5, II-5,II-7,I-4',II-4'-hexahydroxy-(I-3,II-8)-flavonylflavanonol together with two known flavonoids, 6-methyl-4'-methoxyflavone and acacetin. The structures of the compounds were elucidated by spectroscopic methods. The compounds were evaluated for their ability to inhibit PAF receptor binding to rabbit platelets using ^3^H-PAF as a ligand. The biflavonoid and acacetin showed strong inhibition with IC_50_ values of 28.0 and 20.4 µM, respectively. The results suggest that these compounds could be responsible for the strong PAF antagonistic activity of the plant.

## 1. Introduction

Platelet-activating factor (PAF) is a potent glycerophospholipid mediator, participating in a number of physiological responses such as aggregation [1], chemotaxis [2], granule secretion and oxygen radical generation from leukocytes [3,4]. It is also involved in several pathophysiological conditions such as inflammation [5], allergy [6], asthma [7] and thrombosis [8]. Specific receptors for PAF have been reported in a variety of cell membranes, including those from platelets [9]. Therefore, compounds which inhibit the specific binding between PAF and receptors may be useful as leads in the development of therapeutic agents for a variety of inflammation, respiratory, immunological and cardiovascular disorders [10].

*Garcinia nervosa* var. *pubescens* King, locally known in Malaysia as “kandis gajah”, belongs to the Guttiferae plant family. It is a medium-sized tree up to 21 m high and can be easily recognized by its very large and prominently ribbed leaves. The tree produces a yellow or white latex. The species can be found in the lowland forest, especially by rivers [11]. Generally, *Garcinia* species are used in traditional medicine to treat diarrhea, irregular menstruation, earaches, itches, wounds, ulcers, fevers and after childbirth. The fruits of many species are edible [12]. Previous phytochemical investigations on *G. nervosa* revealed the presence of xanthones [13], biflavanoids [14] and isoflavones [15], however, reports on the biological activity of this species is still lacking. In this paper, we report on the strong inhibitory effect (62.1%) of the methanol extract of the leaves of *G. nervosa* var. *pubescens* on PAF binding *in vitro* and the bioassay-guided isolation and structure elucidation of a new biflavonoid from the plant, together with two known flavonoids, and their effect on the binding of ^3^H-PAF to washed rabbit platelets.

## 2. Results and Discussion

The methanol extract of the leaves of *G. nervosa* var *pubescens* was investigated for platelet-activating factor (PAF) receptor binding inhibitory activity on rabbit platelets at a concentration of 18.2 µg/mL. The methanol extract showed an inhibitory effect of 62.1%. The extract was then successively fractionated into ethyl acetate, butanol and methanol fractions and their PAF inhibitor binding was determined. The highest inhibitory activity was obtained with the ethyl acetate fraction (78.0%), as compared to the butanol (47.8%) and methanol fractions (36.2%).

**Figure 1:**
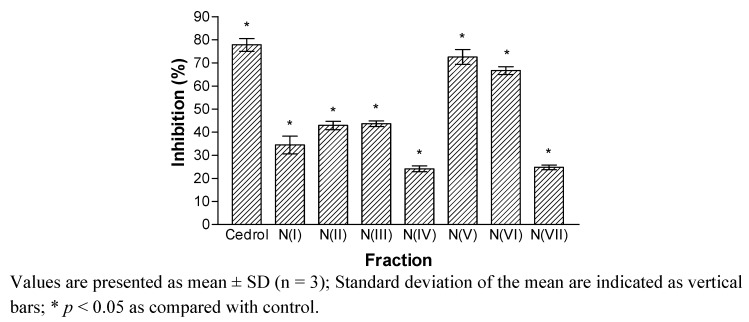
Inhibitory effects of the fractions (I–VII) on the PAF receptor binding to rabbit platelets at 18.2 µg/mL.

The ethyl acetate fraction was chromatographed on a silica gel H column using VLC technique to yield seven fractions (I to VII). Fractions V and VI appeared to demonstrate significant inhibitory activity with inhibition of 72.6 and 66.7%, respectively (Figure 1). 

The results indicated that fraction V and VI contained bioactive compounds with PAF receptor binding inhibitory activity. Fraction V was re-chromatographed on silica gel to give five fractions, NA(1–19), NA(20–59), NA(60–84), NA(85–95) and NA(96–106). Fraction NA(85–95) that showed significant inhibitory effects of more than 60% were further purified to obtain compounds **1** and **2**. Fraction VI was also subjected to silica gel column chromatography to yield fractions NC(1–16), NC(17–32), NC(33–42), NC(43–63) and NC(64–90). Only fraction NC(43–63) exhibited inhibition of more than 60%, while other fractions displayed less than 50% inhibition. Further purification of fraction NC(43–63) yielded compound **3**.

**Figure 2:**
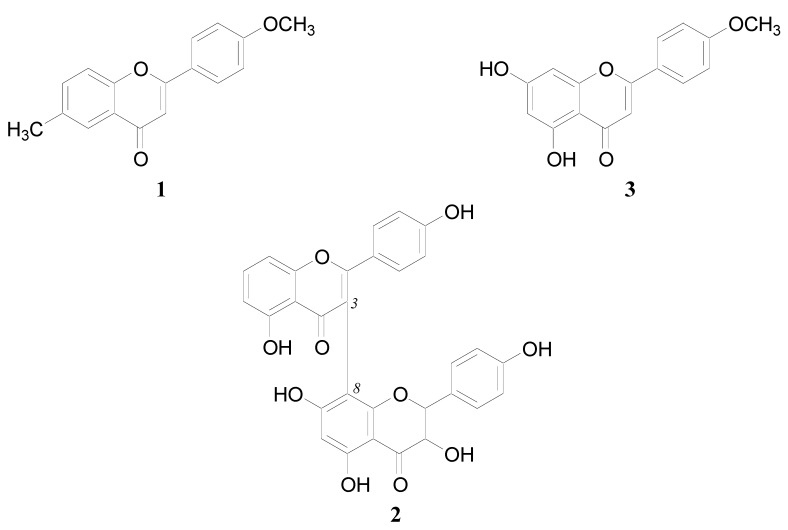
The chemical structures of compounds **1**, **2** and **3**.

Compounds **1**, **2** and **3** were analyzed by spectroscopic techniques to determine their chemical structures. Based on the spectral data and comparison with literature values, compound **1** and **3** were identified as 6-methyl-4'-methoxyflavone [16] and acacetin [17], respectively (Figure 2). Compound **2** is a new compound. Its structure was elucidated by a combination of FAB mass spectrometry, ^1^H-NMR and ^13^C-NMR spectra in combination with 2D-NMR techniques (COSY-45, HMQC and HMBC) (Table 1). The ^13^C-NMR spectrum of **2** showed signals of 30 carbons. Five signals recorded at δ 157.7, 161.5, 161.1, 163.6 and 161.4 were assigned to oxygenated aromatic carbons, while signals for non-oxygenated aromatic carbons were observed in the region δ 98.2–128.5.

**Table 1:** ^1^H-NMR and ^13^C-NMR data for **2** in DMSO-*d_6_*.

			^1^H-^1^H ^b^	^1^H-^13^C ^c^	
C/H	δ_H_	δ_C_^a^	δ_H_	^2^ *J*	^3^ *J*
I-2	-	164.7	-	-	-
I-3	-	101.0	-	-	-
I-4	-	182.7	-	-	-
I-5	-	157.7	-	-	-
I-6	6.65	114.4	-	157.7 (C-I-5)	128.7 (C-I-10)
I-7	7.08	128.5	6.43 (H-I-8), 6.65 (H-I-6)	114.4 (C-I-6)	157.4 (C-I-9)
I-8	6.43	102.2	-	157.4 (C-I-9)	114.4 (C-I-6)
I-9	-	157.4	-	-	-
I-10	-	128.7	-	-	-
I-1'	-	121.8	-	-	-
I-2' & I-6'	7.76	128.5	6.96 (H-I-3' & H-I-5')	-	128.5 (C-I-6' & C-I-2'),161.5 (C-I-4'), 164.7 (C-I-2)
I-3' & I-5'	6.96	115.9	-	161.5 (C-I-4')	115.9 (C-I-5' & C-I-3'),121.8 (C-I-1')
I-4'	-	161.5	-	-	-
II-2	5.75	81.4	-	82.7 (C-II-3), 128.0 (C-II-1')	-
II-3	5.60	82.7	-	81.4 (C-II-2), 197.3 (C-II-4)	-
II-4	-	197.3	-	-	-
II-5	-	161.1	-	-	-
II-6	6.08	98.2	-	161.1 (C-II-5), 163.6 (C-II-7)	100.6 (C-II-8), 104.2 (C-II-10)
II-7	-	163.6	-	-	-
II-8	-	100.6	-	-	-
1I-9	-	162.5	-	-	-
II-10	-	104.2	-	-	-
II-1'	-	128.0	-	-	-
II-2' & II-6'	7.63	128.2	δ 6.71 (H-II-3' & H-II-5')	-	128.2 (C-6' & C-2'),161.4 (C-4')
II-3' & II-5'	6.71	115.8	-	161.4 (C-4')	115.8 (C-5' & C-3')
II-4'	-	161.4	-	-	-

^a^ Assigned by HMQC experiment; ^b^ Assigned by COSY-45 experiment; ^c^ Assigned by HMBC experiment.

The ^1^H-NMR spectrum of **2** exhibited the presence of six hydroxyl groups as six singlets (1H each) in the lower field at δ 8.32, 9.32, 12.23, 12.35, 13.00 and 13.05. The spectrum further showed the presence of doublets (1H each, *J* = 8.0 Hz) at δ 6.43, 6.65 and 7.08 which were assigned to the aromatic protons, H-I-8, H-I-6 and H-I-7, respectively. A singlet for one proton at δ 6.08 was assigned to H-II-6, while doublets at δ 5.75 (1H, *J* = 12.0 Hz) and δ 5.60 (1H, *J* = 12.0 Hz) were allocated to H-II-2 and H-II-3, respectively. A pair of doublets (2H each, *J* = 8.5 Hz) at δ 6.96 and δ 7.76 indicated *ortho*-coupled protons between H-I-5' with H-I-6', and between H-I-2' with H-I-3', respectively. Another pair of *ortho*-coupled doublets (2H each, *J* = 8.5 Hz) at δ 6.71 and δ 7.63 were attributed to H-II-3', 5' and H-II-2', 6', respectively. These data supported that compound **3** was a biflavonoid, composed of a flavone (structure I) and a flavanonol (structure II) unit, linked together at I-3, II-8 with hydoxylated carbon at C-I-3, C-I-5, C-I-4', C-II-5, C-II-7 and C-II-4'.

In the COSY-45 spectrum, the connectivities of the protons were revealed and all protonated carbons were assigned by the HMQC spectrum. In the HMBC spectrum, correlations between the aromatic proton at δ 6.65 (H-I-6) with the carbons at δ 157.7 (C-I-5) and δ 128.7 (C-I-10) were observed. The proton at δ 7.08 (H-I-7) was correlated to the carbons at δ 114.4 (C-I-6) and δ 157.4 (C-I-9), while proton at δ 6.43 (H-I-8) was correlated to the carbons at δ 157.4 (C-I-9) and δ 114.4 (C-I-6). These correlations deduced the 5-hydroxylated ring A structure. The presence of cross peaks between the aromatic protons at δ 7.76 (H-I-2', 6') and the carbons at δ 128.5 (C-I-6', 2'), δ 161.5 (C-I-4') and δ 164.7 (C-I-2), and between the aromatic protons at δ 6.96 (H-I-3', 5') and the carbons at δ 161.5 (C-I-4'), δ 115.9 (C-I-5', 3') and δ 121.8 (C-I-1') supported the 4-hydroxylated ring B structure. The spectrum further showed that proton at δ 5.75 (H-II-2) was correlated to the carbons at δ 82.7 (C-II-3) and δ 128.0 (C-II-1'), while proton at δ 5.60 (H-II-3) was correlated to the carbons at δ 81.4 (C-II-2) and δ 197.3 (C-II-4), implying the presence of a flavanonol unit. The aromatic proton at δ 6.08 (H-II-6) showed correlations with the carbons at δ 161.1 (C-II-5), δ 163.6 (C-II-7), δ 100.6 (C-II-8), δ 104.2 (C-II-10), which supported the 5,7-hydroxylated ring A structure. These correlations also confirmed the involvement of C-II-8 in the interflavonoidic linkage.

Based on these spectroscopic data, compound **2** was characterized as II-3,I-5,II-5,II-7,I-4', II-4'-hexahydroxy-(I-3,II-8)-flavonylflavanonol (Figure 2). This structure was supported by the FAB mass spectrum which exhibited a ion peak [M+H]^+^ at *m/z* 541, suggesting the molecular mass of 540 corresponding to C_30_H_20_O_10_.

Each compound was then tested for PAF receptor binding inhibitor activity at concentration of 18.2 µg/mL. The results showed that compounds **2** and **3** gave strong inhibitory activities of 70.0% and 73.9% respectively, while compound **1** exhibited weak inhibition of 35.7%. Therefore, the inhibitory effects of compounds **2** and **3** at various concentrations were also evaluated to determine their IC_50_ values. The results showed that compounds **2** and **3** displayed concentration-dependent responses, *i.e*., as the concentration of the compound increased, the percentage inhibition increased (Table 2). Probit analysis of these data gave the IC_50_ values for compounds **2** and **3** as 28.0 and 20.4 µM, respectively. The results suggest that compounds **2** and **3** were the major contributors to the PAF receptor binding inhibitor activity of the ethyl acetate fraction of the leaves of *G. nervosa* var. *pubescens*. These values were higher than that of cedrol (10.7 µM), but comparable to the reported value of gingkolide J (54.0 µM) from *Gingko biloba* [18]. Our previous study has also showed that a biflavonoid, amentoflavone, isolated from *Calophyllum inophylloide* exhibited a strong inhibitory effect on PAF receptor binding with an IC_50_ value of 8.3 µM [19]. The results revealed that biflavonoid can represent a new class of natural product which can bind strongly to PAF receptor. The diverse chemical structures of the natural antagonists may suggest that the receptor molecule can accommodate a wide variety of ligand structures, hence, there will be more possibilities of finding new PAF antagonists from natural products. The PAF antagonists have potential to be used as leads in the development of therapeutic agents in a variety of inflammation, respiratory, immunological and cardiovascular disorders.

**Table 2:** Inhibitory effects of compound **2** and **3** on the PAF receptor binding to rabbit platelets at various concentrations and their IC_50_ values.

Compound	Concentration (μg/mL)/% Inhibition				IC_50_ (μM)
	18.2	9.1	4.5	1.8	(95% confidence intervals)
**2**	72.2	45.3	38.1	23.6	28.0 (22.0–37.1)
**3**	72.6	37.8	17.5	6.4	20.4 (17.4–24.4)
**Cedrol**	75.2	65.9	56.2	47.8	10.7 (4.1–16.8)

## 3. Experimental

### 3.1. General Procedures

Radiolabeled PAF (1-*O*-^3^H-octadecyl-2-acetyl-*sn*-glycero-3-phosphocholine, 125 Ci/mmol) was purchased from Amersham (Buckinghamshire, UK). Unlabeled PAF and cedrol were obtained from Sigma Chemical Co. (St. Louis, MO, USA). Bovine serum albumin (BSA) was purchased from Boehringer Mannheim Co. (Mannheim, Germany). Other chemicals were obtained from BDH Laboratory Supplies (Poole, UK). All the reagents and solvents used in this study were of analytical grade. Vacuum liquid chromatography (VLC) was performed on silica gel H (Merck, 10–40 µm), column chromatography (CC) on silica gel 60 (Merck, 230–400 mesh). Preparative TLC used was precoated Merck silica gel 60 F254 plates. Melting points were determined by using a Electrothermal model 9100 hot stage melting point apparatus and were uncorrected. The UV spectra were obtained from Shimadzu 1800 UV-Vis Spectrophotometer. NMR data were measured on a 500 MHz NMR spectrometer (Varian, CA, USA) with deuterated solvents. Molecular weights of the compounds were recorded by EIMS (70 eV) and FABMS (glycerol matrix) using a VG 70-SE mass spectrometer. Radioactivity was measured by a liquid scintillation counter (LSC) (Packard Tri-Carb, models 2100TR, Hamburg, Germany).

### 3.2. Plant Material

The leaves of *Garcinia nervosa* var. *pubescens* were collected from Pasoh, Negeri Sembilan, Malaysia and was identified by Norseha Ayop, a taxonomist from the Forest Research Institute of Malaysia (FRIM), Kepong, Malaysia. A voucher specimen (FRI 43362) was deposited at the Herbarium of FRIM.

### 3.3. Bioassay-Guided Isolation

Air-dried leaves (1.1 kg) of *Garcinia nervosa* var. *pubescens* were ground and extracted with methanol (3 L) using a Soxhlet apparatus. After evaporation of the solvent under reduced pressure, the methanol extract (112 g) was refluxed successively with three different solvents to give 45 g of ethyl acetate, 21 g of butanol and 31 g of methanol fractions. Each fraction was tested for PAF receptor inhibitor binding activity at a concentration of 18.2 µg/mL. The ethyl acetate fraction which showed the highest inhibitory activity, was selected for further purification. The ethyl acetate fraction (20 g) was fractionated by VLC on silica gel H eluted with a gradient solvent system of hexane-CHCl_3_, CHCl_3_-EtOAc and EtOAc-MeOH. Fractions of 250 mL were collected and combined into seven fractions, (I to VII), according to their TLC profiles. After tested with PAF receptor binding assay, the active fractions (V and VI) were rechromatographed on silica gel 60 (230–400 mesh) eluted with CHCl_3_ and methanol to give several active fractions. Further purification of the active fractions yielded compounds **1** (5 mg), **2** (13 mg) and **3** (8 mg). Structural elucidation of the compounds was performed by spectroscopic methods (1D- and 2D-NMR, IR, UV and MS) and the PAF antagonistic activity of each compound was determined.

*II-3,I-5,II-5,II-7,I-4',II-4'-hexahydroxy-(I-3,II-8)-flavonylflavanonol* (**2**). A solid yellow amorphous. UV λ_max_ (MeOH) nm: 290, 338. FABMS *m/z* (rel. int.): 541 (M+H^+^, 20). ^1^H-NMR (500 MHz, DMSO-*d_6_*) δ ppm: 5.60 (1H, d, *J* = 12.0 Hz, H-II-3), 5.75 (1H, d, *J* = 12.0 Hz, H-II-2), 6.08 (1H, s, H-II-6), 6.43 (1H, d, *J* = 8.0 Hz, H-I-8), 6.65 (1H, d, *J* = 8.0 Hz, H-I-6), 6.71 (2H, d, *J* = 8.5 Hz, H-II-3' and H-II-5'), 6.96 (2H, d, *J* = 8.5 Hz, H-I-3' and H-I-5'), 7.08 (1H, d, *J* = 8.0 Hz, H-I-7), 7.63 (2H, d, *J* = 8.5 Hz, H-II-2' and H-II-6'), 7.76 (2H, d, *J* = 8.5 Hz, H-I-2' and H-I-6'), 8.32, 9.32, 12.23, 12.35, 13.0, 13.05 (s, 6 × OH). ^13^C-NMR (125 MHz, DMSO-*d_6_*) δ ppm: 81.4 (C-II-2), 82.7 (C-II-3), 98.2 (C-II-6), 100.6 (C-II-8), 101.1 (C-I-3), 102.2 (C-I-8), 104.2 (C-II-10), 114.4 (C-I-6), 115.8 (C-II-3' and C-II-5'), 115.9 (C-I-3' and C-I-5'), 121.8 (C-I-1'), 128.0 (C-II-1'), 128.2 (C-II-2' and C-II-6'), 128.5 (C-I-7, C-I-2' and C-I-6'), 128.7 (C-I-10), 157.4 (C-I-9), 157.7 (C-I-5), 161.1 (C-II-5), 161.4 (C-II-4'), 161.5 (C-I-4'), 162.5 (C-II-9), 163.6 (C-II-7), 164.7 (C-I-2), 182.7 (C-I-4), 197.3 (C-II-4).

### 3.4. Preparation of Samples for PAF Assay

Each sample was dissolved in dimethyl sulfoxide (DMSO) and ethanol (1:1). Then, the stock solutions were diluted with normal saline to give final concentrations of 200 μg/mL. The final concentration of DMSO in reaction mixture was fixed at 0.2% to avoid interference with the receptor binding studies. Reaction mixture with saline and 0.2% DMSO in saline was used as control. The final concentration of each sample in the reaction mixture was 18.2 μg/mL.

Tris-tyrode buffer (10 mM, pH 7.0) was used as media for binding studies. ACD solution (0.15 M trisodium citrate, 0.075 M citric acid, pH 5.2) was used as anticoagulant. Buffer A (20% ACD solution, 60% K_2_HPO_4_ buffer, 20% sodium citrate, pH 6.8) and buffer B (50 K_2_HPO_4_ buffer, 0.1 g bovine serum albumin (BSA), pH 7.0) were used for washing of platelets. Six volumes of blood were collected from rabbit (New Zealand White) marginal ear veins directly into one volume of ACD solution. The procedure was under approval of the Animal Ethics Committee of the Universiti Kebangsaan Malaysia (approval; no. FSKB/2007/Juriyati/10-July/192). The blood was centrifuged at 270 × *g* for 10 min at room temperature, and the top platelet-rich plasma was removed carefully. The latter was further centrifuged at 500 × *g* for 15 min. The platelet pellets were washed two times by centrifugation at 500 × *g* (15 min) in buffer A followed by 50 × *g* (10 min) in buffer B. The top whitish layer was removed and centrifuged at 500 × *g* (15 min) to obtain the platelets. The final platelet concentration was adjusted to 3 × 10^8^ platelets/mL.

### 3.5. PAF Receptor Binding Inhibitor Assay

The assay was carried out according to the method described by Jantan *et al.* [19] and cedrol was used as a positive control. The reaction mixtures consisted of 200 μL of washed rabbit platelet suspension, 25 μL of ^3^H-PAF (2.0 nM) with or without unlabeled PAF (2.0 μM) and 25 μL of sample or control solution were incubated at room temperature for 1 h. The free and bound ligands were separated by filtration technique using a glass microfiber filter in cell harvester. The radioactivity was measured by liquid scintillation counter. The difference between total radioactivities of bound ^3^H-PAF in the absence and the presence of excess unlabeled PAF is defined as specific binding of the radiolabeled ligand. Percentage inhibition of the sample was obtained according to the following equation:


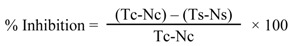


where Tc = Total binding of control; Ts = Total binding of sample; Nc = Nonspecific binding of control; Ns = Nonspecific binding of sample.

### 3.6. Statistical Analysis

The percentage inhibition values are reported as the means ± SD of three separate experiments. The IC_50_ values were determined by using Probit computer program with 95% confidence intervals. 

## 4. Conclusions

The present study indicates that *G. nervosa* may contain promising therapeutic agents for PAF-related diseases. Further studies are necessary to elucidate the mechanisms behind their anti-inflammatory effects.
